# Chromatin Remodeling Enzyme Snf2h Is Essential for Retinal Cell Proliferation and Photoreceptor Maintenance

**DOI:** 10.3390/cells12071035

**Published:** 2023-03-28

**Authors:** Andrea Kuzelova, Naoko Dupacova, Barbora Antosova, Sweetu Susan Sunny, Zbynek Kozmik, Jan Paces, Arthur I. Skoultchi, Tomas Stopka, Zbynek Kozmik

**Affiliations:** 1 Laboratory of Transcriptional Regulation, Institute of Molecular Genetics of the Czech Academy of Sciences, 142 20 Prague, Czech Republic; 2 Laboratory of Genomics and Bioinformatics, Institute of Molecular Genetics of the Czech Academy of Sciences, Videnska 1083, 142 20 Prague, Czech Republic; 3 Department of Cell Biology, Albert Einstein College of Medicine, 1300 Morris Park Ave., Bronx, NY 10461, USA; 4 Biocev, First Faculty of Medicine, Charles University, Prumyslova 595, 252 50 Vestec, Czech Republic; 5 Research Unit for Rare Diseases, Department of Paediatrics and Inherited Metabolic Disorders, First Faculty of Medicine, Charles University and General University Hospital in Prague, 128 08 Prague, Czech Republic

**Keywords:** Snf2h, Smarca5, retina, photoreceptors, cell cycle, apoptosis

## Abstract

Chromatin remodeling complexes are required for many distinct nuclear processes such as transcription, DNA replication, and DNA repair. However, the contribution of these complexes to the development of complex tissues within an organism is poorly characterized. Imitation switch (ISWI) proteins are among the most evolutionarily conserved ATP-dependent chromatin remodeling factors and are represented by yeast Isw1/Isw2, and their vertebrate counterparts Snf2h (Smarca5) and Snf2l (Smarca1). In this study, we focused on the role of the *Snf2h* gene during the development of the mammalian retina. We show that *Snf2h* is expressed in both retinal progenitors and post-mitotic retinal cells. Using *Snf2h* conditional knockout mice (*Snf2h* cKO), we found that when *Snf2h* is deleted, the laminar structure of the adult retina is not retained, the overall thickness of the retina is significantly reduced compared with controls, and the outer nuclear layer (ONL) is completely missing. The depletion of Snf2h did not influence the ability of retinal progenitors to generate all the differentiated retinal cell types. Instead, the Snf2h function is critical for the proliferation of retinal progenitor cells. Cells lacking Snf2h have a defective S-phase, leading to the entire cell division process impairments. Although all retinal cell types appear to be specified in the absence of the Snf2h function, cell-cycle defects and concomitantly increased apoptosis in *Snf2h* cKO result in abnormal retina lamination, complete destruction of the photoreceptor layer, and consequently, a physiologically non-functional retina.

## 1. Introduction

In eukaryotes, the chromosomal DNA is compacted in a highly organized nucleoprotein structure called chromatin that presents a barrier to most cellular processes [1]. The nucleosome is the basic structural unit of chromatin and is composed of four histone cores (H2A, H2B, H3, and H4) around which 147 bp of DNA are wrapped [1]. Various ATP-dependent chromatin remodeling complexes can reposition nucleosomes through the energy released via ATP hydrolysis [2,3]. Among the chromatin remodeling ATPases, the imitation switch (ISWI) family is highly conserved during evolution [4,5,6,7]. Mammals have two ISWI homologs—Snf2h (sucrose nonfermenting 2 homolog; also known as Smarca 5) and Snf2l (sucrose nonfermenting 2-like; also known as Smarca 1). Both proteins are present in complexes with a diverse array of noncatalytic subunits and are therefore able to promote many biological functions, including DNA replication, DNA repair, transcriptional repression or activation, and maintenance of chromosome structure [8,9,10,11,12,13,14].

Snf2h is known to function as the catalytic ATPase in at least five distinct complexes in mammalian cells, including WICH, CHRAC, ACF, RSF, and NoRC (reviewed in [3,15]). The presence of Snf2h within these complexes and the interactions of these complexes with other partners result in the targeting of Snf2h for particular biological functions in chromatin. For example, Snf2h is recruited to double-strand breaks by sirtuin protein SIRT6 to promote DNA break repair [10]. The lack of SIRT6 and Snf2h impairs chromatin remodeling, increasing sensitivity to genotoxic damage and recruitment of downstream factors such as 53BP1 [10]. Snf2h also plays a major role in organizing arrays of nucleosomes adjacent to the binding sites for the architectural chromatin factor CTCF and acts to promote CTCF binding on DNA [8]. Several studies have focused on the in vivo function of Snf2h ATPases and ISWI-containing complexes [6,7,16,17,18,19,20,21,22,23]. However, the simultaneous presence of Snf2h in distinct multicomponent complexes dedicated to unrelated nuclear functions makes it harder to interpret the molecular mechanisms underlying the observed pathological states in the context of a tissue or organism [17,22,24].

There are seven major cell types in the mammalian retina that are generated from a common pool of multipotent retinal progenitor cells (RPCs) [25,26,27]. Each cell type is born in a defined order [26,28,29,30] and plays a specific role in visual perception [31,32]. Ganglion cells (GCs) arise first, around embryonic day 11 (E11) in mice, and are the only retinal type whose axons project to the brain [31,33,34]. Simultaneously, the propagation of other early-born retinal neurons occurs. Horizontal cells (HCs), amacrine cells (ACs), and cone photoreceptors are produced with the highest peak at E14 [35,36,37,38]. Thereafter, later-born cells begin to form, namely, bipolar cells (BCs), Müller glia cells (MCs), and rod photoreceptors, whose generation increases shortly after birth [26,36,38,39]. The process of retinal differentiation in mice is finished at postnatal day 14 (P14) when the eyes open [40]. Mature retinal neurons and glia are arranged in three main layers—ganglion cell layer (GCL), inner nuclear layer (INL), and outer nuclear layer (ONL) [27]. With respect to the entire eye, the GCL is located closest to the lens and is particularly occupied by GCs [36]. The interneurons (HCs, ACs, and BCs), primarily associated with the transmission of information throughout the retina, are placed together with MCs, ensuring retinal homeostasis, in the INL [27,36,41]. The ONL, the outermost retinal region, is formed by photoreceptors [36]. Cones and rods are specialized sensory neurons that absorb photons of light and activate the process of photo-transduction [42]. Postnatally, murine photoreceptors express different types of opsin proteins—rhodopsin gene expression is characteristic of rod photoreceptors, while S-opsin (short-wavelength opsins) and M-opsin (medium-wavelength opsins) expression characterize cone photoreceptors. Photoreceptors are highly metabolically active and require a stable cellular environment; otherwise, the cell morphology or physiology is disrupted [43,44]. A number of studies investigated the role of transcription factors in photoreceptor development [45,46,47,48,49,50,51,52,53,54,55,56]. In contrast, the regulatory molecules affecting photoreceptor cell maintenance during embryonic and postnatal stages remain largely unknown. It is well established that in order to generate retinal diversity, while maintaining appropriate cell numbers, a proper balance between cell proliferation and differentiation of progenitor cells is required [57,58]. These events are regulated by various extrinsic and intrinsic cues, from which the transcription factors seem to be the most relevant (reviewed in [58,59]). Recent epigenetic studies show that the chromatin-modifying or remodeling mechanisms are also important for retina development and maintenance; however, it is unknown how Snf2h, a key component in chromatin remodeling complexes, is involved in the process [60,61,62,63,64,65]. Here, we studied the role of the Snf2h during mouse retinal development and differentiation. Since *Snf2h*-deficient mice die at the peri-implantation stage due to the growth arrest of trophectoderm and inner cell mass [22], we performed conditional gene targeting using the floxed allele of *Snf2h* [17] and mRx-Cre active in RPCs from E9.0 onwards [66]. 

## 2. Materials and Methods

### 2.1. Mouse Lines

For the retina-specific inactivation of *Snf2h*, mRx-Cre [66] and Snf2h^fl/fl^ [17] mice were used. 

### 2.2. Tissue Collection and Histology

Mouse embryos were harvested from timed pregnant females. The morning of the vaginal plug was determined as embryonic day 0.5 (E0.5). The embryos and eyes of postnatal mice were fixed in 4% formaldehyde in PBS (*w*/*v*) overnight at 4 °C. The next day samples were washed with cold PBS and incubated in 70% ethanol. Subsequently, samples were dehydrated, embedded in paraffin blocks, and sectioned. Horizontal sections of 5 µm were prepared, stored at 4 °C, and used for up to one month. 

### 2.3. Immunohistochemistry

Paraffin sections were deparaffinized and rehydrated. The epitope retrieval was performed for 20 min in a citrate buffer (10 mM, pH 6.0) at 98 °C in a pressure cooker. For immunofluorescent analysis, the sections were washed three times in PBT, blocked with 10% BSA in PBT (*w*/*v*) for one hour, and incubated overnight with primary antibody at 4 °C (diluted in 1% BSA/PBT). The sections were washed three times in PBT, incubated for 45 min at room temperature with a secondary antibody, washed three times with PBT, and covered with DAPI/PBS (1 µg/mL) for 10 min. After washing in PBT, the sections were mounted into Mowiol (488; Sigma, Munich, Germany). For the immunohistological analysis, the dewaxed sections were washed three times in PBT, treated with 1.5% H_2_O_2_ in 10% methanol in PBS for 25 min, washed again with PBT, blocked with 10% BSA/PBT for one hour, and incubated with primary antibody overnight at 4 °C. The applied primary antibody was detected with a biotinylated secondary antibody (Vector Laboratories, Burlingame, CA, USA) and visualized with Vectastain ABC Elite kit and ImmPACT DAB substrate (all Vector Laboratories). The primary antibodies used for immunohistochemistry are listed in Table S1. The standard histological staining of paraffin sections using hematoxylin and eosin (H&E) was also performed. At least three different embryos from at least two different litters were analyzed with each staining.

### 2.4. EdU Incorporation

Timed pregnant females were injected intraperitoneally with 13 µg per g body weight of 5-ethynyl-2′-deoxyuridine (Edu; Thermo Fisher Scientific, Waltham, MA, USA) and sacrificed after 1 h or 24 h. Whole embryos were processed identically as described above. The acquired paraffin sections were deparaffinized, rehydrated, and washed three times in PBT. The sections were incubated in proteinase K/TE buffer (20 µg/mL) at 37 °C for 10 min and washed with PBT. The sections were treated with 0.3% H_2_O_2_ in methanol for 20 min at room temperature and washed three times in PBT and incubated with Click-iT Edu Imaging kit (AlexaFluor azide, Click-iT Edu reaction buffer; CuSO_4_, Click-iT Edu buffer additive) for 1 h. After rinsing, the sections were incubated with DAPI/PBS (1 µg/mL) for 10 min, washed in PBT, and mounted into Mowiol (4–88; Sigma-Aldrich, Munich, Germany). 

### 2.5. Quantification of Marker-Positive Cells

The quantification analysis of different retinal cell types, including apoptotic cells and cell-cycle analysis, was performed via the manual counting of marker-positive cells per central retinal section. In case an eye was removed from the individual (postnatal stages), the eye was precisely oriented into the paraffin block, and the central region was verified using a magnifier. Only those sections that were cut through the optic nerve were taken into account. The number of marker-positive cells per whole retinal section (GCs and HCs) or per defined retinal area in the central retinal part (ACs, BCs, MCs, rods, cones, apoptotic cells, and proliferating cells) was counted and normalized to wild-type controls. For a single eye, a minimum of six sections was used; for each genotype, a minimum of four individual retinae was analyzed. Statistical significance was assessed using Student’s *t*-test.

### 2.6. Quantitative RT-PCR (qRT-PCR)

Differentially expressed genes related to the p53 pathway in wild-type and *Snf2h* deficient retinal cells were established in P0 eyes. Postnatal retinae were dissected from the eye, separately from retinal pigment epithelium (RPE) and lens. Total RNA was isolated with a Trizol Reagent (Life Technologies, Carlsbad, CA, USA). Random-primed cDNA was generated from 500 ng total RNA using a SuperScript VILO cDNA Synthesis Kit (Life Technologies). Six different individuals originating from three litters were used for tissue dissection and subsequent analysis, for both wild-type and *Snf2h*-deficient cells. qRT-PCR reactions were run in a LightCycler 480 Instrument (Roche, Basel, Switzerland) using a 5 µL reaction mixture of DNA SYBR Green I Master (Roche) according to the standard manufacturer’s protocol. Analysis was performed on the replicates of six different individual samples per genotype, run in triplicate. Crossing point (Cp) values were calculated with LightCycler 480 Software (Roche) using the second-derivate maximum algorithm. The average Cp values of all the biological and technical replicates were normalized using the Cp values of housekeeping genes *Gapdh*, *Ubb*, and *Actb*. The statistical significance of the change in mRNA expression was calculated using a two-tailed Student’s-test in Microsoft Excel. Finally, the change in mRNA expression was presented as the ratio of mRx-Cre; Snf2h^fl/fl^ to wild-type retinae on a log2 scale. Primer sequences are listed in Table S2.

### 2.7. RNA-Seq

Raw data were obtained from Gene Expression Omnibus (GEO) under accession number GSE87064 and used for re-analysis [67]. RNA-seq reads were preprocessed with FASTX tool kit v0.0.11 to remove short and low-quality reads. The reads were aligned to the mm10 genome using HISAT2 V2.1.0 [68]. 

## 3. Results

### 3.1. Conditional Deletion of Snf2h Disrupts Retinal Morphology

**Figure 1:**
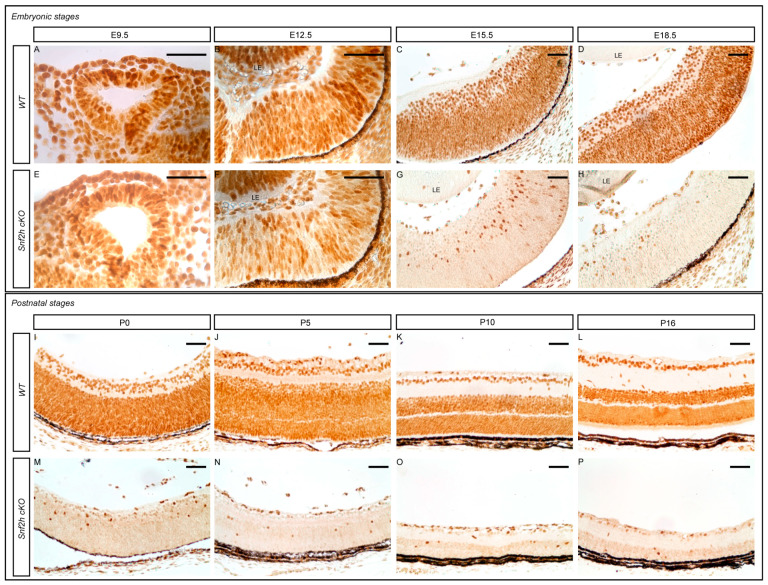
*Snf2h* expression during retina development. Immunohistochemistry staining of wild-type and mRx-Cre; Snf2h^fl/fl^ (*Snf2h* cKO) mice was carried out with anti-Snf2h specific antibodies in different embryonic and postnatal stages. *Snf2h* started to be expressed in early embryonic stages and was maintained in wild-type individuals throughout embryonic development, and in adulthood in both retinal progenitors and differentiated retinal cells (**A**–**D**,**I**–**L**). A decrease in Snf2h levels was first observed in E12.5 conditionally mutant retinae (**A**,**B**,**E**,**F**). During the following embryonic stages, a rapid reduction in Snf2h-positive cells in *Snf2h* cKO eye sections occurred (**G**,**H**), and only a few Snf2h-positive cells were found in *Snf2h* cKO at the postnatal stage (**M**–**P**). LE–lens. Scale bars: 50 µm.

To determine *Snf2h* expression during mouse retinal development, we performed immunohistochemistry staining stages E9.5 to P18. Snf2h was detected in all RPCs of the optic cup at E9.5 and subsequently in all RPCs and differentiated retinal cells during the later embryonic stages (Figure 1A–D). After birth, strong *Snf2h* expression was detectable throughout the entire retina, independently of the cell type (Figure 1I–L). Snf2h functions in multiple protein complexes [69]. We, therefore, examined gene expression changes in Snf2h and its interacting partners during retinal development with publicly available RNA-seq data [67]. The CORUM database was used to identify the components of the complexes [70]. *Snf2h* was remarkably upregulated during P0 and P3 (Figure S1). Interestingly, the expression of genes encoding ACF, CHRAC, WICH, B-WICH, cohesin, All-1, and Dnmt3b including complex members was also increased (Figures S1–S3). 

**Figure 2:**
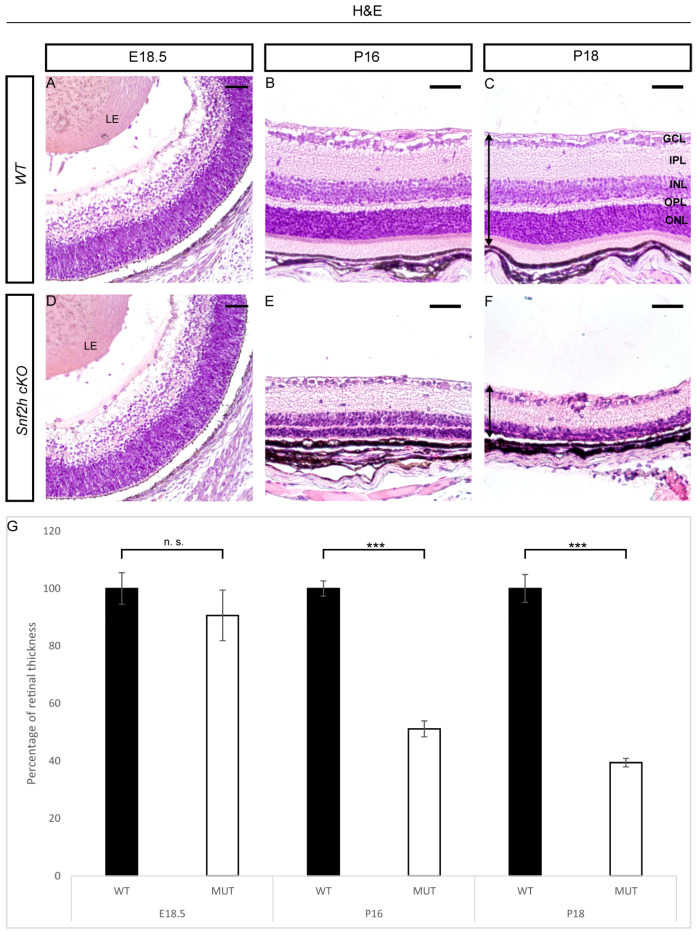
Morphology of *Snf2h*-deficient retina. Hematoxylin and eosin staining of embryonic and postnatal retinal sections of wild-type and *Snf2h* cKO was performed to display the retinal morphology. At the time of fully differentiated retinal cell types (P18), *Snf2h* cKO had dramatically reduced retinal thickness compared with controls (**C**,**F**). The outer retinal segment of *Snf2h* cKO mice was completely missing at P18 (**F**). *Snf2h*-deficient mice maintained the appropriate retinal structure until P16, although the outer nuclear layer (ONL) was almost indistinguishable (**B**,**E**). Differences in the thickness of the retina were not significant until birth (**A**,**D**,**G**). The time course of the retinal thinning is shown in the graph (**G**). The arrows show the range from where the retinal thickness was measured (**C**,**F**). GCL—ganglion cell layer, INL—inner nuclear layer, ONL—outer nuclear layer, IPL—inner plexiform layer, OPL—outer plexiform layer, and LE—lens. Error bars indicate standard derivation; *p*-values were calculated using Student’s *t*-test (*n* = 3). *** *p* < 0.001, n.s. = not significant. Scale bars: 50 µm.

In order to investigate the function of the *Snf2h* gene during retinal development, we conditionally inactivated *Snf2h* in RPCs and their progeny by crossing mRx-Cre mice with Snf2h^fl/fl^ mice. The mRx-Cre retinal driver is active in RPCs from E9.0 onwards [66], which corresponds with the onset of retinal neurogenesis. We analyzed the extent of Snf2h depletion in the retina of mRx-Cre Snf2h^fl/fl^ mice (referred to as *Snf2h* cKO) via immunohistochemistry. The first conspicuous reduction in Snf2h immunoreactivity was observed at E12.5 (Figure 1F), i.e., at a time when the early-born retinal cell types are already being generated [36]. It is therefore likely that many GCs, HCs, ACs, and cones are established in the presence of functional Snf2h protein in the *Snf2h* cKO embryo. During the following embryonic stages examined, the number of retinal cells expressing Snf2h rapidly decreased, and from E15.5 onwards, almost all the remaining cells lacked the Snf2h protein (Figure 1G,H,M–P). Therefore, some early-born and almost all late-born retinal cells developed in the absence of the Snf2h function. To investigate the phenotypic consequences of Snf2h deficiency, we first analyzed the retinal morphology with hematoxylin–eosin (H&E) staining. We did not find any obvious difference between wild-type and *Snf2h* cKO mice in the early embryonic stages (E9.5–E15.5). The retinal thickness of *Snf2h* cKO was comparable to wild-type control at E16.5 (Figure 2A,D), and no significant difference was observed until E18.5 (Figure 2G). After birth, the thickness of the *Snf2h*-deficient retina gradually decreased, and at P18, the retina was reduced by 60% compared with the wild-type control (Figure 2G). In contrast, *Snf2h* heterozygote mice (genotype mRx-Cre;Snf2h^fl/+^) had normal retinal thickness and morphology even at postnatal week 50 (PW 50, Figure S4), indicating that a single functional allele of *Snf2h* is sufficient for normal retina development. The reduction in retinal thickness in *Snf2h* cKO was accompanied by the selective loss of the outer retinal segment, including ONL and the outer plexiform layer (OPL) (Figure 2F). It is of note that retinal lamination was preserved until P16, although the ONL was poorly distinguishable (Figure 2E). The GCL and INL, along with the inner plexiform layer (IPL), were retained but were thinner than the wild-type control (Figure 2C,F). 

### 3.2. Snf2h Is Required for Photoreceptor Maintenance and Visual Perception of Mice

**Figure 3:**
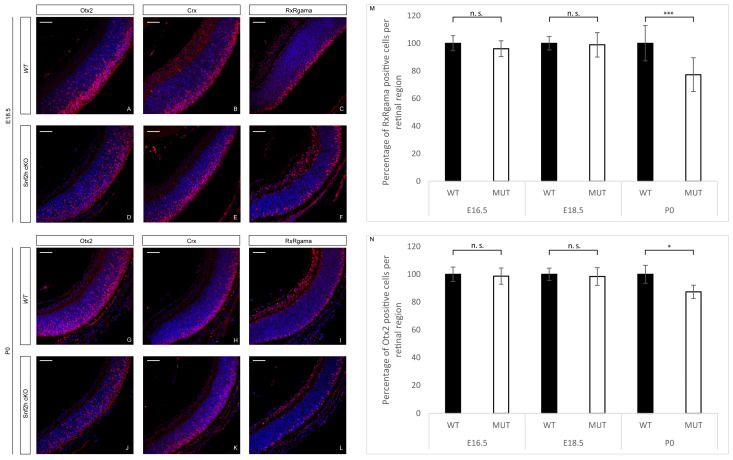
Photoreceptor specification in *Snf2h* cKO. To screen the photoreceptor development, a variety of different photoreceptor-specific markers were used; among them, Otx2, Crx, and Rxrγ were chosen for the embryonic immunofluorescent analysis. The immunostained retinal sections showed no significant differences between *Snf2h* cKO mice and controls at E18.5 (**A**–**F**). However, there was a rapid decrease in Rxrγ- and Otx2-positive cells in *Snf2h*-deficient mice immediately after birth (**G**–**N**). Error bars indicate standard derivation; *p*-values were calculated using Student’s *t*-test (*n* = 3). * *p* < 0.05, *** *p* < 0.001, n.s. = not significant. Scale bars: 50 µm.

Considering that the outermost layer of the retina, which is missing in adult *Snf2h*-deficient mice, is formed by photoreceptors, we analyzed differentiation of cones and rods in *Snf2h* cKO mice during embryonic and postnatal stages. To follow photoreceptor specification and maturation, a set of antibodies directed against Otx2, Crx, and Rxrγ was used for photoreceptor mapping during embryonic stages. No significant differences in the immunoreactivity for these photoreceptor markers were observed in *Snf2h* cKO at E18.5 compared with controls (Figure 3A–F). At P0, we observed an approximately 25% reduction in photoreceptors in *Snf2h* cKO (Figure 3G–N). 

**Figure 4:**
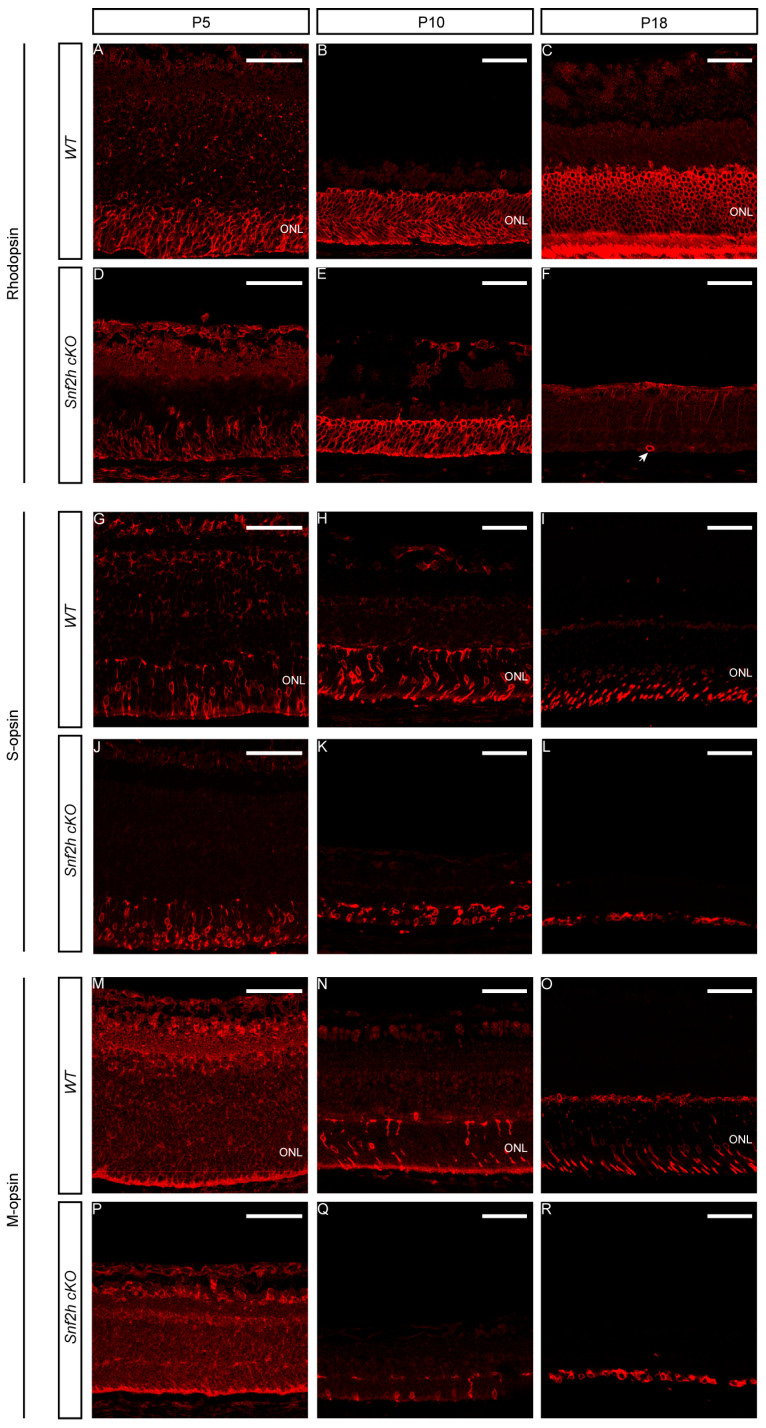
Photoreceptor maintenance in *Snf2h* cKO during postnatal stages. Specific opsin markers were used to determine the photoreceptor state in *Snf2h* cKO after birth. Rhodopsin detects the rod photoreceptors S-opsin (the short-wavelength cone photoreceptors) and M-opsin (the medium-wavelength cones). Except for M-opsin, which was not expressed until P8, opsin expression was mapped from P5 to P18 (**A**–**R**). At P10, all opsins were expressed in *Snf2h*-deficient retinae, although the ONL layer was significantly reduced in thickness compared with controls (**B**,**E**,**H**,**K**,**N**,**Q**). At P18, no rhodopsin-positive photoreceptors were present in *Snf2h* cKO, and only scattered rhodopsin-positive residues were identified in the entire retinal section (**F**, indicated by an arrow). At P18, the differences between *Snf2h* cKO and controls in S-opsin (**I**,**L**) and M-opsin (**O**,**R**) expression were conspicuous, but not as pronounced as the loss of rhodopsin expression. Nevertheless, the shape of cone photoreceptors appeared abnormal. Scale bars: 50 µm.

At postnatal stages, we used different opsin markers specific for cones or rods to follow photoreceptor maturation based on their onset, the level of expression, and sub-cellular localization. Rhodopsins are expressed throughout the entire length of the outer retinal segment, whereas M-opsins are preferentially located dorsally, and S-opsins are located ventrally [55,71]. Since the mouse retina is rod-dominated, the prevailing opsin is rhodopsin, which was first detectable in control mice at P5 (Figure 4A). In *Snf2h* cKO mice, the process of rod photoreceptor maturation appeared to occur normally, although the signal strength of immunostaining was weaker than wild-type controls (Figure 4D). An approximately normal level of rhodopsin expression was detected in *Snf2h*-deficient retina even at P10 (Figure 4E). Shortly thereafter, the expression of rhodopsin was extinguished, and only sparse rhodopsin-positive cell residues were present at P18 (Figure 4F). A similar result was obtained via immunostaining for cone photoreceptors. We used two mouse cone-opsin-specific markers, S-opsin (short wavelength) and M-opsin (medium wavelength). At P5, the expression of S-opsin was weaker in *Snf2h* cKO mice compared with controls (Figure 4G,J). At P10, the *Snf2h* deficient retina still retained some S-opsin-positive cells, but compared with wild-type, their numbers were reduced (Figure 4H,K). Finally, at P18, the *Snf2h*-deficient cones lacked their characteristic shape and S-opsin positive residues were accumulated just below the INL (Figure 4I,L). The M-opsin staining at P18 showed a pattern overall similar to that of S-opsin (Figure 4R). Since the main decline in photoreceptor development occurred between P10 and P18, we hypothesized that the key event leading to photoreceptor damage is the opening of the eye at P14. The animals were analyzed at P13, when the eyes are still closed, and at P14, after eye opening. A noticeable difference in the thickness of *Snf2h* cKO retinas between the P13 and P14 stages was observed (Figure S5). Immunodetection for rhodopsin, M- and S-opsin confirmed the significant deterioration of the morphology of the outer photoreceptor segment between stages P13 (Figure S6) and P14 (Figure S7) in *Snf2h* cKO mice. The outer photoreceptor segments were extremely shortened, lost their orientation, and spread within the outermost retinal layer. Our data, therefore, suggest that an excess of light at the eye-opening stage is not a leading factor of photoreceptor damage in *Snf2h* cKO. Instead, the loss of photoreceptors is regulated by intrinsic cues and is gradual. 

### 3.3. All Other Retinal Cell Types Are Specified in the Absence of Snf2h Function

**Figure 5:**
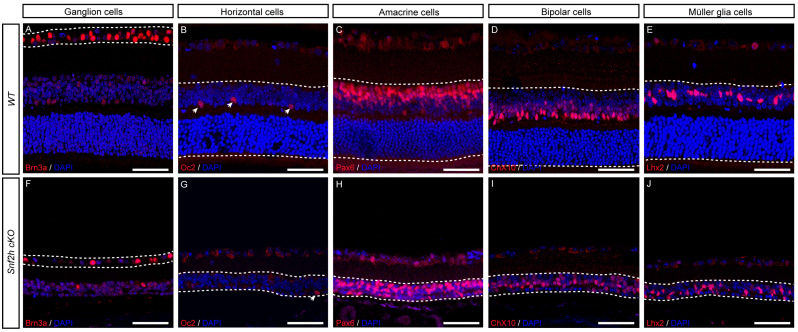
All cell types in GCL and INL are present in the *Snf2h*-deficient retina. GCs in the GCL were detected using anti-Brn3a antibodies in the P18 retina (**A**,**F**). The interneurons in INL of P18 (**B**,**D**,**E**,**G**,**I**,**J**) or P22 (**C**,**H**) were identified by the following molecular markers: Oc2 (HCs), Pax6 (ACs), Chx10 (BCs), and Lhx2 (MCs). Scale bars: 50 µm.

To investigate whether the Snf2h function is not restricted to photoreceptors but is also required for the generation and/or maintenance of other retinal cell types, we performed immunohistochemistry labeling for specific retinal markers of each cell type. We found that GCs (Brn3a-positive cells, Figure 5A,F), HCs (Oc2-positive cells, Figure 5B,G), ACs (Pax6-positive cells, Figure 5C,H), BCs (Chx10-positive cells, Figure 5D,I), and MCs (Lhx2-positive cells, Figure 5E,J) were present in Snf2h-deficient retina. Combined, our data indicate that *Snf2h* is not necessary for the specification of these cell types in the mature mouse retina.

### 3.4. Loss of Snf2h Causes Cell-Cycle Defects and Increased Apoptosis

**Figure 6:**
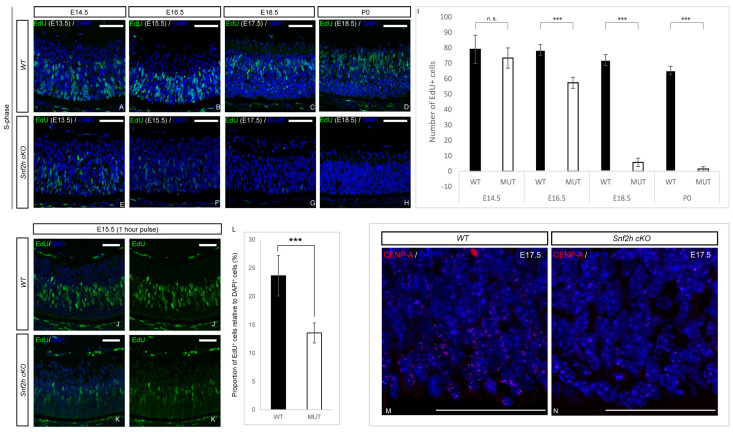
Cell-cycle analysis of *Snf2h*-deficient retinal cells. The retinal proliferation was analyzed via EdU incorporation. Pregnant females were intraperitonally injected with EdU 24 h prior to sacrifice at indicated stages. The number of EdU^+^ cells in wild-type and *Snf2h* cKO mice was comparable until E14.5. The differences first appeared at E16.5—the number of EdU^+^ cells was decreased by ~25% in *Snf2h*-deficient mice compared with controls. Subsequently, at E18.5 and after birth, almost no EdU^+^ cells were detectable in *Snf2h* cKO, in contrast with the massive proliferation rate in wild-type retinae (**A**–**I**). Pregnant females were injected with EdU one hour prior to sacrifice. The proportion of EdU^+^ cells/DAPI^+^ cells was reduced in *Snf2h*-deficient retina at E15.5 (**J**, **J**’,**K**, **K**’,**L**). Expression of CENP-A (centromere protein A) at E17.5. CENP-A is essential for centromere localization and proper chromosomal segregation. The CENP-A antibody staining was widespread in wild-type retinae at E17.5 (**M**), whereas in *Snf2h*-depleted retinae, only a few CENP-A-positive cells were found (**N**), indicating impaired chromosomal segregation in *Snf2h* cKO retinal cells. Error bars indicate standard derivation; *p*-values were calculated using Student’s *t*-test (*n* = 4). *** *p* < 0.001, n.s. = not significant. Scale bars: 50 µm.

Next, we sought to determine whether the decreased retinal cell numbers were due to poor RPC expansion or the initiation of intense apoptosis, or both. To analyze retinal cell proliferation, we examined EdU incorporation during embryonic retina development following a 24 h chase period. The EdU incorporation at P0 revealed a dramatic reduction in the number of EdU-positive cells in *Snf2h* cKO—almost no EdU^+^ cells were detectable in contrast to controls, where the proliferation was still high and concentrated in the INL (Figure 6D,H,I). Such an extreme loss of proliferating cells explains the sharp reduction in retinal thickness after birth. A major proliferation defect was already observed during embryonic development. The EdU labeling of wild-type and *Snf2h* cKO retinae significantly differed at E16.5, and the difference increased with each consecutive embryonic day (Figure 6A–H,I). The reduction in EdU-positive cells in the *Snf2h*-deficient retina was already detectable at E15.5 following a brief 1 h pulse (Figure 6J–L). Very similar data were obtained using cyclin D1 immunofluorescent labeling (Figure S8), which not only reflected the S-phase but also the G1/S transition. Combined, these results indicate that Snf2h is necessary for the proper initiation and progress of DNA replication in retinal cells, which is consistent with previous in vitro studies [72,73,74,75]. Next, we used an anti-CENP-A (centromere protein A) antibody recognizing the H3 histone variant that is incorporated into centromeric nucleosomes and is essential for centromere localization and chromosomal segregation. The overall signal was substantially decreased in *Snf2h* cKO mice, in contrast with wild-type animals at E17.5 (Figure 6M,N). 

**Figure 7:**
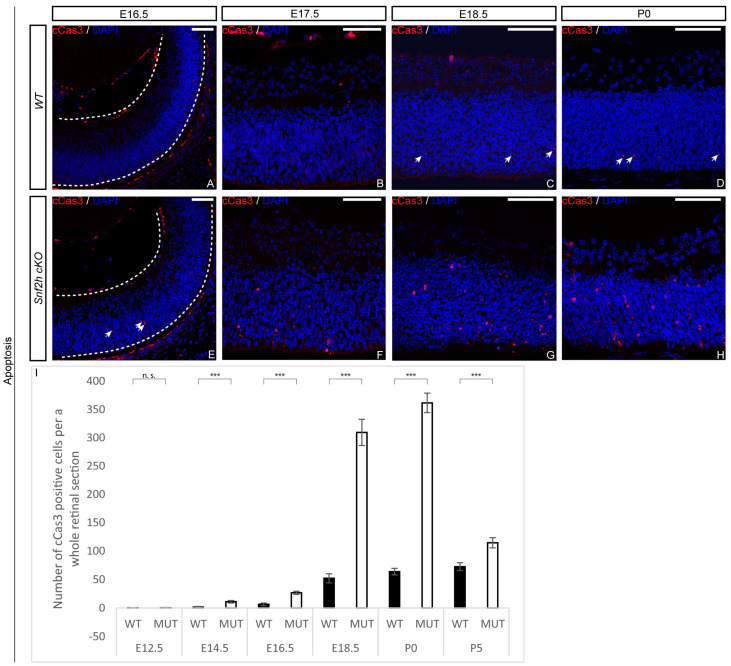
Loss of Snf2h in developing retina is accompanied by massive apoptosis. The number of apoptotic cells in embryonic and postnatal retinae was detected using the anti-cleaved caspase-3 (cCas3) antibody. The differences between *Snf2h* cKO and controls first appeared at E14.5 (**I**) and gradually increased during later embryonic stages (**A**–**C**,**E**–**G**,**I**). The peak of apoptosis in *Snf2h*-deficient mice was at P0 (**D**,**H**,**I**). The number of cCas3-positive cells in *Snf2h*-deficient retinae decreased during the postnatal stages; nevertheless, it remained significantly higher at P5 compared with wild-type controls (**I**). Error bars indicate standard derivation; *p*-values were calculated using Student’s *t*-test (*n* = 4). *** *p* < 0.001, n.s. = not significant. White arrows indicate cCas3-positive cells. Scale bars: 50 m.

**Figure 8:**
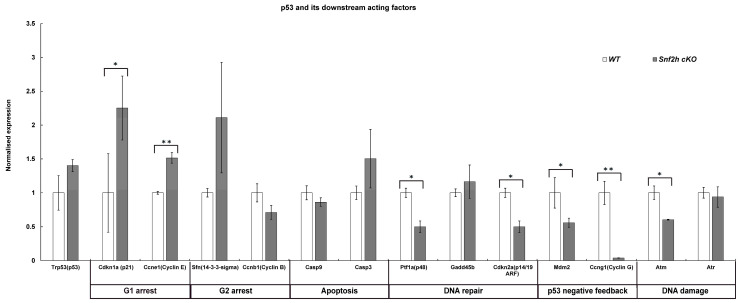
Quantification of mRNA expression of p53 downstream acting factors at P0 assessed with qRT-PCR. Whole retinae from P0 wild-type and *Snf2h* cKO eyes were dissected, subjected to RNA isolation, and processed using qRT-PCR. A total of 3 retinas were tested. Error bars indicate standard derivation; *p*-values were calculated using Student’s *t*-test (*n* = 3). * *p* < 0.05, ** *p* < 0.01.

This result suggests that in *Snf2h* cKO retinal cells, the chromosomes are not able to attach to the spindle apparatus and are therefore not separated. Next, we investigated whether the dramatic decrease in retinal cell numbers was also partly due to increased apoptosis. It was indeed likely that the DNA damage in *Snf2h* cKO led to chromosomal instability and an increased rate of programmed cell death. We first analyzed the apoptosis using an anti-cleaved caspase-3 (cCas3) antibody at E16.5, and in correlation with pronounced proliferation defects, the number of apoptotic cells also increased (Figure 7A,I). A dramatic increase in cCas3-positive cells was observed in *Snf2h* cKO compared with controls at P0 (Figure 7D,H,I). It should be noted that both poor replication and an increase in apoptosis were not unique to any specific retinal layer but observed in almost the entire retina. The p53 pathway is often activated upon DNA damage, leading to increased apoptosis. To determine whether the p53 pathway was activated in *Snf2h*-deficient retinae, we analyzed the expression of the pathway-related genes using qRT-PCR (Figure 8). We focused on genes directly controlled by p53, namely *Cdkn1a* (p21), *Cdkn2a* (Arf), *Atm*, *Atr*, *Mdm2*, *Ptf1a* (p48), *Casp3*, *Casp9*, *Ccna2* (cyclin A), *Ccnb1* (cyclin B), *Ccne1* (cyclin E), and *Ccng1* (cyclin G). These genes encode the proteins involved in various functions: Some are necessary for entering the next cell-cycle phase, some act as checkpoints, and others change their expression levels upon the programmed cell death or directly inhibit p53 function. The qRT-PCR confirmed the increased expression of the *Trp53* gene (p53) in *Snf2h* cKO retinae. In addition, the levels of cyclin inhibitor p21 were elevated. This result suggests that the *Snf2h*-depleted retinal cells are arrested in the G1-phase of the cell cycle. The altered levels of cyclin E and cyclin B furthermore indicated possible irregularities during S-phase progression, whereas the reduced levels of both Atm and Atr mRNAs in *Snf2h* cKO retinae showed impairment in cell-cycle checkpoints. Severe downregulation of cyclin G mRNA levels in *Snf2h*-deficient retinae indicated impaired p53 negative feedback loop, a condition that may result in the constitutive activation of the p53 pathway. Finally, we compared the expression levels of caspase-3 and caspase-9 genes in wild-type and *Snf2h* cKO. Although both genes are expressed during apoptosis, only caspase-3 mRNA levels were upregulated in the mutant retinae. 

Taken together, our analyses demonstrate cell-cycle defects, the activation of the p53 pathway mRNA targets, and increased apoptosis in *Snf2h* cKO retinae.

## 4. Discussion

The Snf2h protein acts as a chromatin remodeler. Here, through conditional gene targeting and detailed phenotypic analysis, we elucidated the function of the *Snf2h* gene during the development, differentiation, and maturation of the mouse retina. Considering that Snf2h serves as a catalytic subunit (ATPase) and is incorporated into several distinct ISWI chromatin remodeling complexes (ACF, WICH, CHRAC, RSF, and NoRC), the loss of Snf2h likely results in broad impairment of the chromatin dynamics and organization. Snf2h acts as the main executive component of these complexes, whereas the other subunits enhance and direct diverse processes such as DNA replication, DNA repair, recombination, and transcription [15,76,77]. Defects observed here in *Snf2h* cKO might be due to the Snf2h function in any of these processes alone or due to a combination of defects in several of them. Our data indicated that the *Snf2h*-deficient retina exhibited impaired progenitor cell expansion. Based on EdU incorporation and the immunofluorescent staining of proliferation markers, we identified a dominant defect in the S-phase of the cell cycle (the process of DNA replication). When *Snf2h* cKO mice were compared with controls, the first remarkable decline in S-phase progression manifested itself at E16.5, i.e., soon after the common peak of production of early-born retinal cells [35,36,37,38]. During later embryonic development, the replication activity gradually decreased in *Snf2h* cKO and was minimal at birth. In contrast, in wild-type retinae, proliferation continued approximately up to P8 [30,78,79]. Such a rapid loss of DNA replication was previously observed in the *Snf2h*-depleted Purkinje cells of the mouse cerebellum [17]. Our qRT-PCR results provided further support for aberrant S-phase progression and specifically pointed to the G1/S transition. The increased levels of p21 and cyclin E mRNAs in *Snf2h*-depleted retinal cells indicated cell-cycle arrest in G1. It is therefore likely that the extremely rapid decline in DNA replication in embryonic retinal cells is due to the combination of both factors. The importance of Snf2h for DNA replication during the cell cycle was previously shown by several in vitro studies [72,73,74,75]. These studies were focused on the specific aspects of the S-phase, and all established the key role of Snf2h-containing complexes during heterochromatin replication. In addition, the Snf2h function is required in the early S-phase [75], in which the actively transcribed genes within euchromatin are replicated [80]. We propose that the process of cell division is impaired in the *Snf2h* cKO retinae. Heterochromatin, whose replication is primarily disrupted, is incorporated into the pericentromeric region. This region is characterized as a condensed, transcriptionally repressed chromatin part, necessary for genome stability, chromosome pairing, and segregation [81,82,83]. Perpelescu et al. [84] established a key role for the RSF complex, composed of Rsf1 (remodeling and spacing factor 1) and Snf2h, in maintaining a proper centromere structure via the stabilization of the centromere protein A histone variant (CENPA). Our CENP-A analysis indicates that the *Snf2h*-depleted mitotic chromosomes do not contain functional centromeres. It is interesting that Alvarez-Saavedra et al. [17] listed *HJURP* (Holliday junction recognition protein) as a gene downregulated in the *Snf2h*-deficient cerebellum. HJURP was recently identified as a protein involved in the localization of CENP-A into the centromere and thereby is critical for centromere formation and maintenance [85,86,87,88]. If the division of the cell nucleus is indeed not properly carried out, then the *Snf2h*-deficient retinal cells likely carry an abnormal number of chromosomes. Hereby, we can conclude that the *Snf2h* gene is required for the proliferation of RPCs. In the absence of the Snf2h function, several cell-cycle processes occur incorrectly. 

The loss of Snf2h in the retina leads to massive apoptosis demonstrated by an increased number of cleaved caspase-3-positive cells from E16.5 onwards, with the highest peak at P0. Caspase-3 activates the extrinsic apoptotic pathway, whereas caspase-9, whose levels were not significantly changed in the *Snf2h* cKO retinae, activates the intrinsic apoptotic pathway. We speculate that the loss of Snf2h and the activation of the p53 pathway influences only the extrinsic apoptotic pathway whose effector caspase is caspase-3. 

Given that all retinal cell types are developed from a common pool of retinal progenitor cells, the population of which depletes with proceeding retinogenesis, it is not surprising that the proliferation defects in *Snf2h* cKO cause retina abnormalities in young adult mice. More remarkable is, however, that the degeneration predominantly affects photoreceptor cells. The reason for this is currently unclear. Cones and rods occupy the outermost layer of the retina, and their basic function and morphology are similar despite their different birth order during retinogenesis. Cones are generated along with the early-born retinal neurons, whereas rods are classified as later-born cells [36].

The Cre driver used here, the mRx-Cre BAC transgenic line, is an early retina-specific deleter active from E9.0 [66]. In our previous study using this Cre line, we were able to efficiently deplete Pax6 already by E10.5 [89] and Meis1/Meis2 by E14.5 [90]. However, due to the Snf2h protein stability, the conspicuous depletion of the Snf2h protein was observed by E12.5, and little to no Snf2h immunoreactivity was present only by E15.5, which is still much earlier than the P0 stage by which we observed the elimination of the extremely stable polycomb proteins [65]. Considering that the Snf2h depletion in *Snf2h* cKO started at E12.5, many cones were still generated in the presence of Snf2h. This was, however, not the case for late-born rods. Originally, we hypothesized that the birth order of each retinal cell type would play a role in the resulting phenotype of *Snf2h* cKO mice. We assumed that the late-born cell types that develop in the absence of the Snf2h function (BCs, MGs, and rod photoreceptors) would be much more affected than the early-born neurons. Our results indicated, however, that the birth order did not generally correlate with the magnitude of the loss of a particular retinal cell type. GCs, ACs, MGs, and BCs were present in adult mice, whereas the photoreceptors had a tendency to disappear completely. Based on marker analysis during embryonic stages (Otx, Crx, Blimp, and Rxrγ immunoreactivity), we concluded that the photoreceptors in *Snf2h* cKO were correctly specified and were generated in numbers comparable to wild-type controls. The small loss in photoreceptor numbers occurring before birth was associated with insufficient proliferation and increased apoptosis. Overall, the process of photoreceptor degeneration appeared to be gradual and not directly triggered by external stimuli such as light. Moreover, on closer observation, we found that the photoreceptor collapse could also be caused by damage in synaptogenesis. In the case in which the photoreceptor cells lose contact with either interneurons or retinal pigment epithelium, a subsequent degenerative process is initiated [91,92,93,94]. In the case of *Snf2h* cKO, retinal degeneration occurs early and is fairly rapid. When retinal degeneration occurs, MCs can activate a gliosis process, which leads to the formation of a glial scar containing a range of auxiliary materials [95,96]. This in turn acts as a protection against further neuronal deprivation [97,98]. Such a protective mechanism in the *Snf2h*-deficient retina could perhaps contribute to the maintenance of GCL and INL, including the relevant cell types, even in aging mice (at PW 50). 

## 5. Conclusions

Our analysis of *Snf2h*-deficient mice revealed that Snf2h controls the expansion of the pool of RPCs by safeguarding the cell-cycle progress. In addition, Snf2h appears to be critically required for photoreceptor maintenance in the postnatal mouse retina. Since at the molecular level, Snf2h is a catalytic subunit of several distinct multicomponent complexes dedicated to unrelated nuclear processes, it is unlikely that all the defects observed in *Snf2h* cKO are due to a single Snf2h complex. Further studies aimed at the functional characterization of other components of Snf2h-containing complexes are needed in order to dissect their specific roles in retina growth, maturation, and maintenance.

## Data Availability

The data presented in this study are available on request from the corresponding author.

## Supplementary Materials

The following supporting information can be downloaded at: https://www.mdpi.com/article/10.3390/cells12071035/s1. Figure S1: Expression of Snf2h and its interacting partners during retinal development; Figure S2: Expression of components of ALL-1 supercomplex and PRC1 complex during retinal development. Figure S3: Expression of centromere complex components during retinal Development; Figure S4: Morphology of heterozygous mice at postnatal week 50 (PW50); Figure S5: Retinal morphology of Snf2h cKO at P13 and P14; Figure S6: Photoreceptor characterization at P13 (before eye opening); Figure S7: Photoreceptor characterization at P14 (after eye opening); Figure S8: Expression of cyclin D1 in Snf2h cKO during embryonic and postnatal stages; Table S1. List of primary antibodies used for immunohistochemistry; Table S2: List of primers used for qRT-PCR.
