# Supplementary material for: Chromatin Remodeling Enzyme Snf2h Is Essential for Retinal Cell Proliferation and Photoreceptor Maintenance
Source: Cells. 2023 Mar 28;12(7):1035. doi: 10.3390/cells12071035 (PMC10093269; doi:10.3390/cells12071035)
Supplement: Supplementary file 1 [file cells-12-01035-s001.zip › cells-2252104-supplementary.pdf]

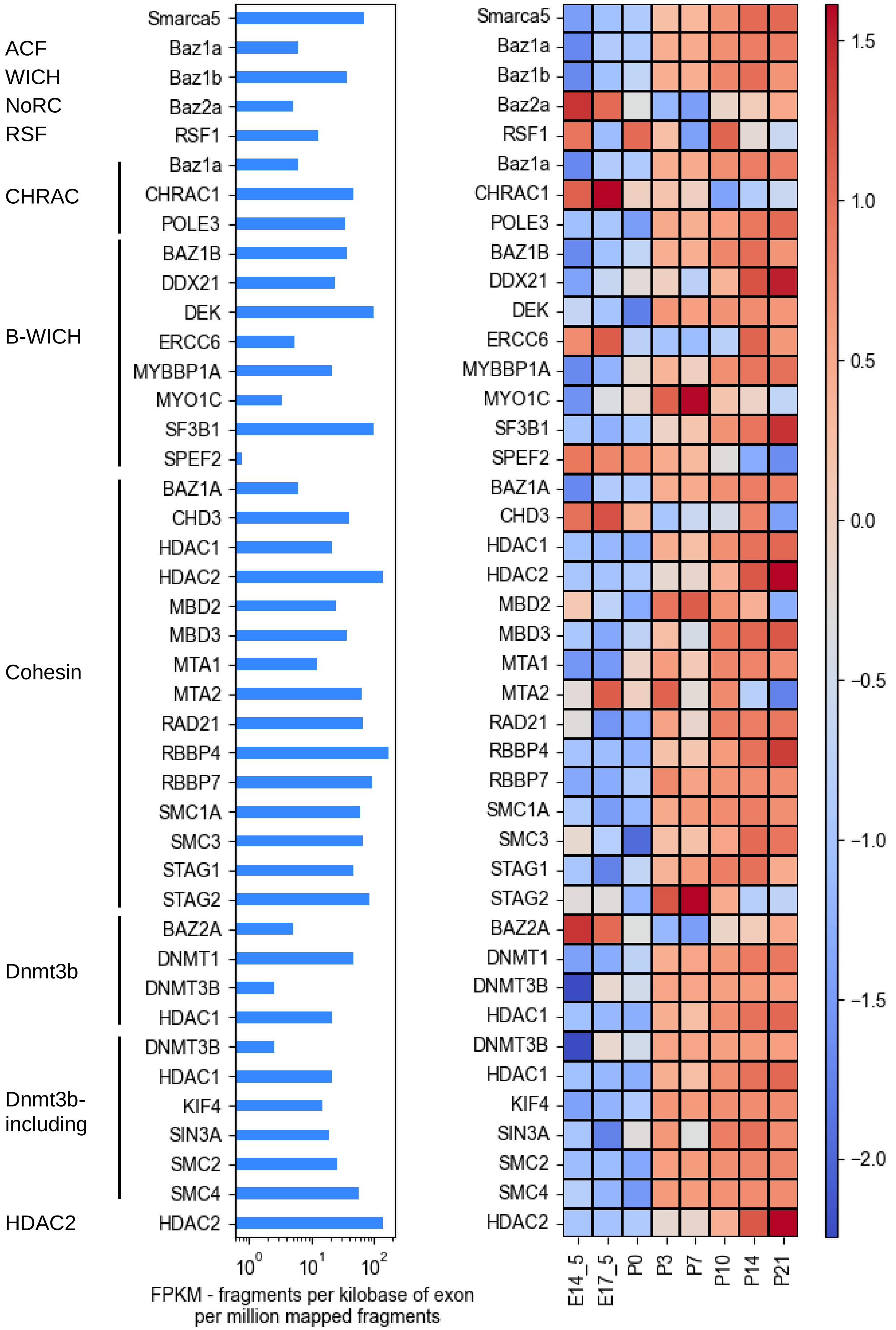

Supplementary Figure S1. Expression of Snf2h and its interacting partners during retinal development.

Heatmap showing expression of components of ACF,WICH,NoRC, RSF, CHRAC, B-WICH, Cohesin, Dnmt3b, Dnmt3b-including, and HDAC2 complexes in retina at E14.5, E17.5, P0, P3, P7, P10, P14, and P21 (GSE87064 (Aldiri et al., 2017)). The left graph indicates FPKM.

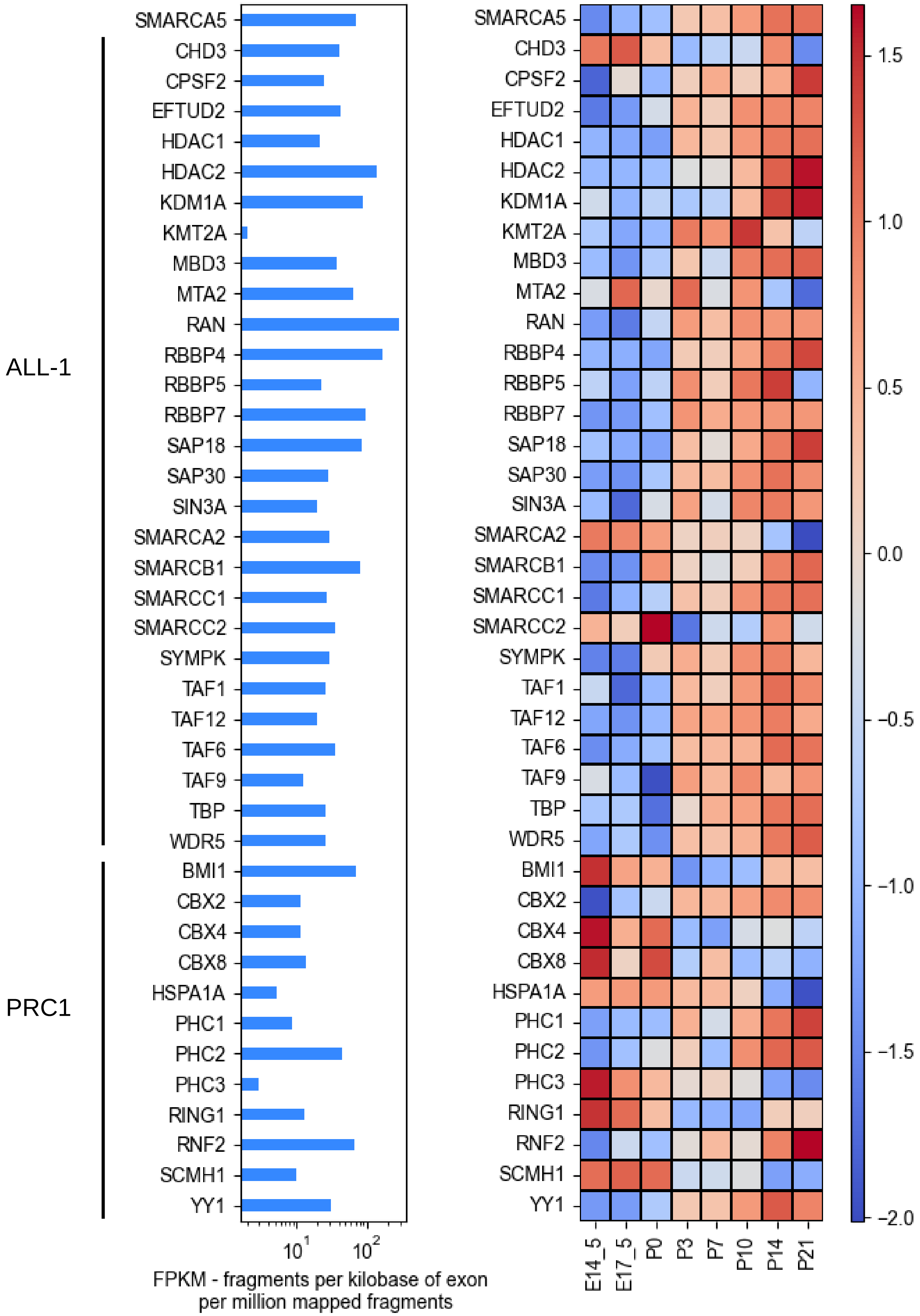

Supplementary Figure S2. Expression of components of ALL-1 supercomplex and PRC1 complex during retinal development.

Heatmap showing expression of components of ALL-1 supercomplex and PRC1 complex in retina at E14.5, E17.5, P0, P3, P7, P10, P14, and P21 (GSE87064 (Aldiri et al., 2017)). The left graphindicates FPKM.

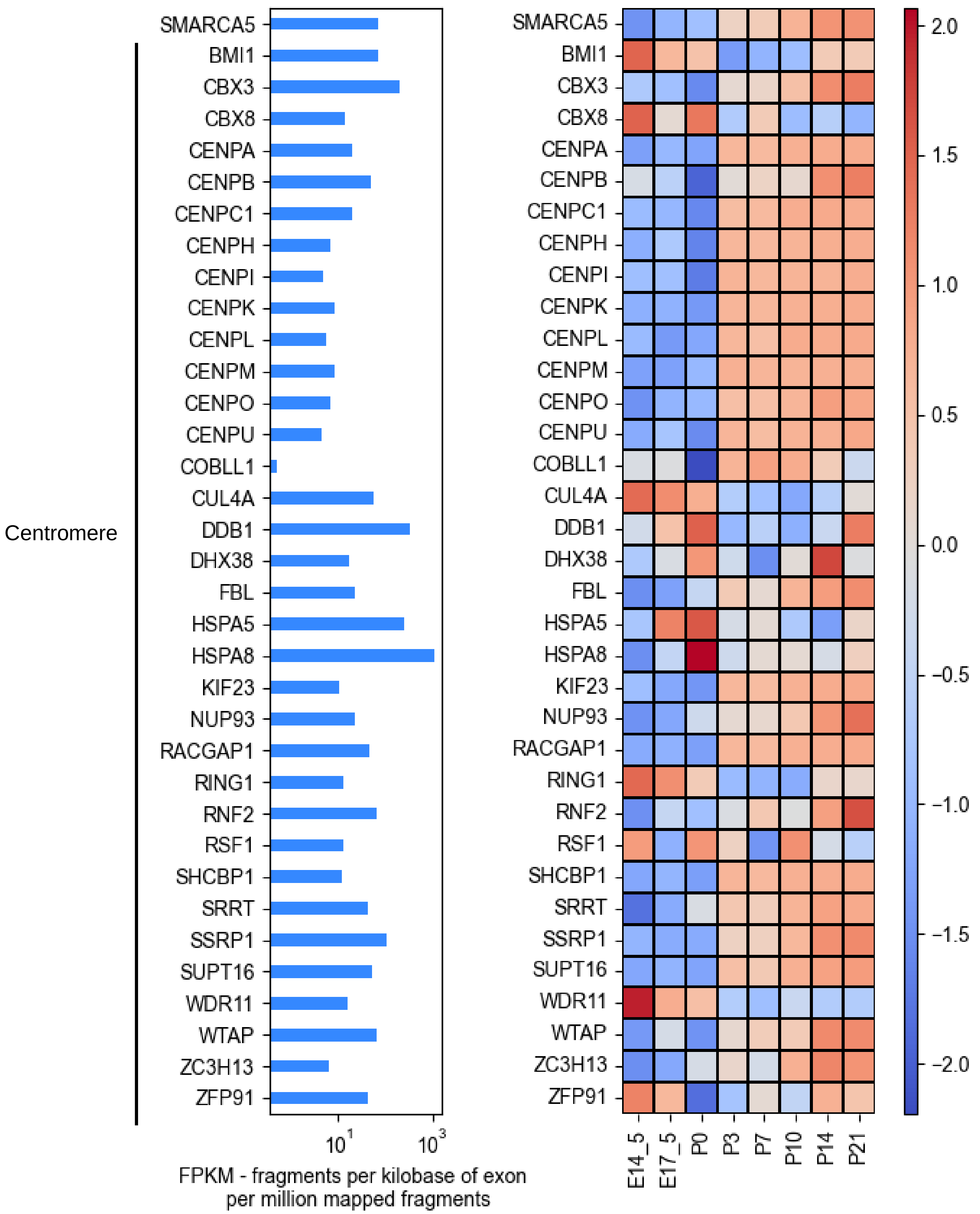

Supplementary Figure S 3. Expression of centromere complex components during retinal development.

Heatmap showing expression of components of centromere complex in retina at E14.5, E17.5, P0, P3, P7, P10, P14, and P21 (GSE87064 (Aldiri et al., 2017)). The left graph indicates FPKM.

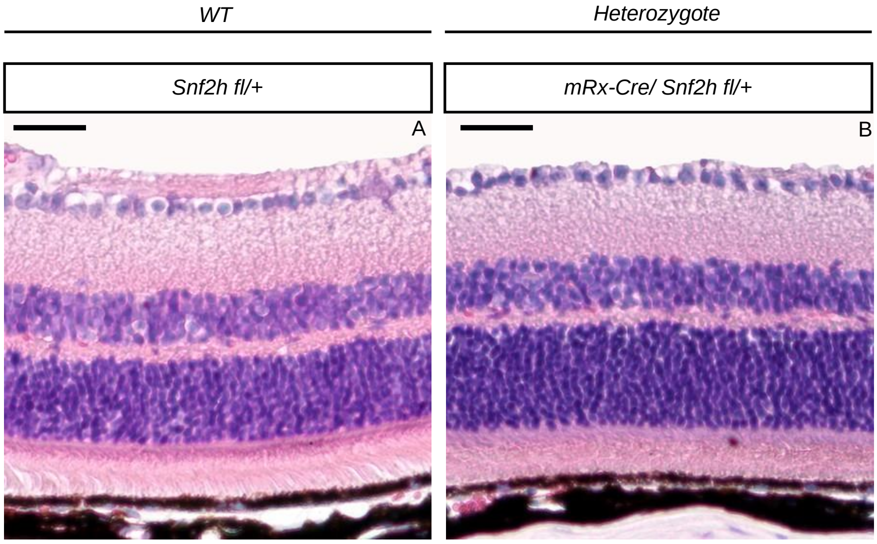

Supplementary Figure S4. Morphology of heterozygous mice at postnatal week 50 (PW50).

Hematoxylin and eosin staining of wild-type (A) and mRx-Cre/ Snf2h$^{fl/+}$ (B) mice did not manifest obvious differences between the two genotypes. One active allele of the Snf2h gene is thus sufficient for the maintenance of an apparently normal gross retinal morphology.

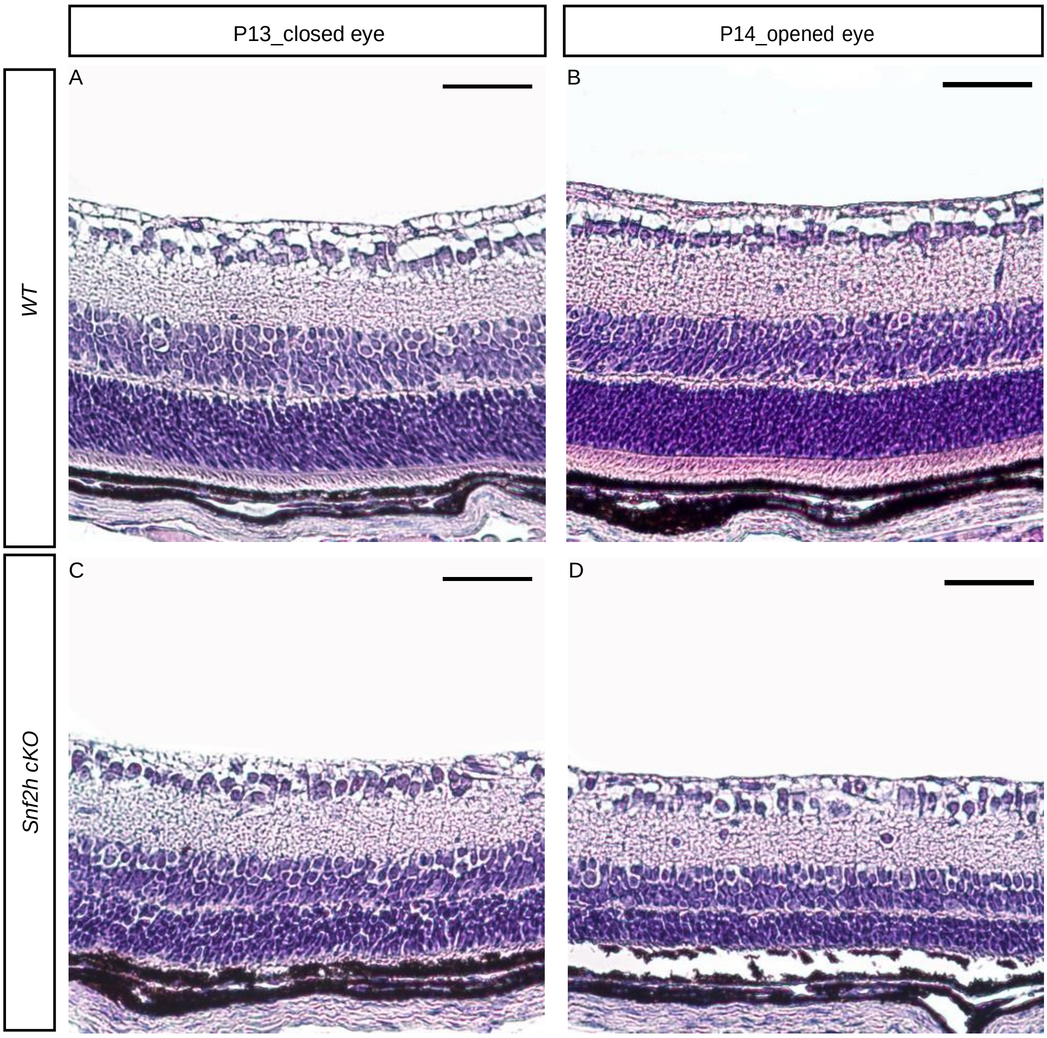

Supplementary Figure S5. Retinal morphology of Snf2h cKO at P13 and P14.

Hematoxylin-eosin staining of postnatal retinal sections at postnatal day 13 (P13) and day 14 (P14) of wild-type and Snf2h cKO. The retinal section of Snf2h cKO at P14 (D) was clearly thinner compared with the same genotype at P13 (C).

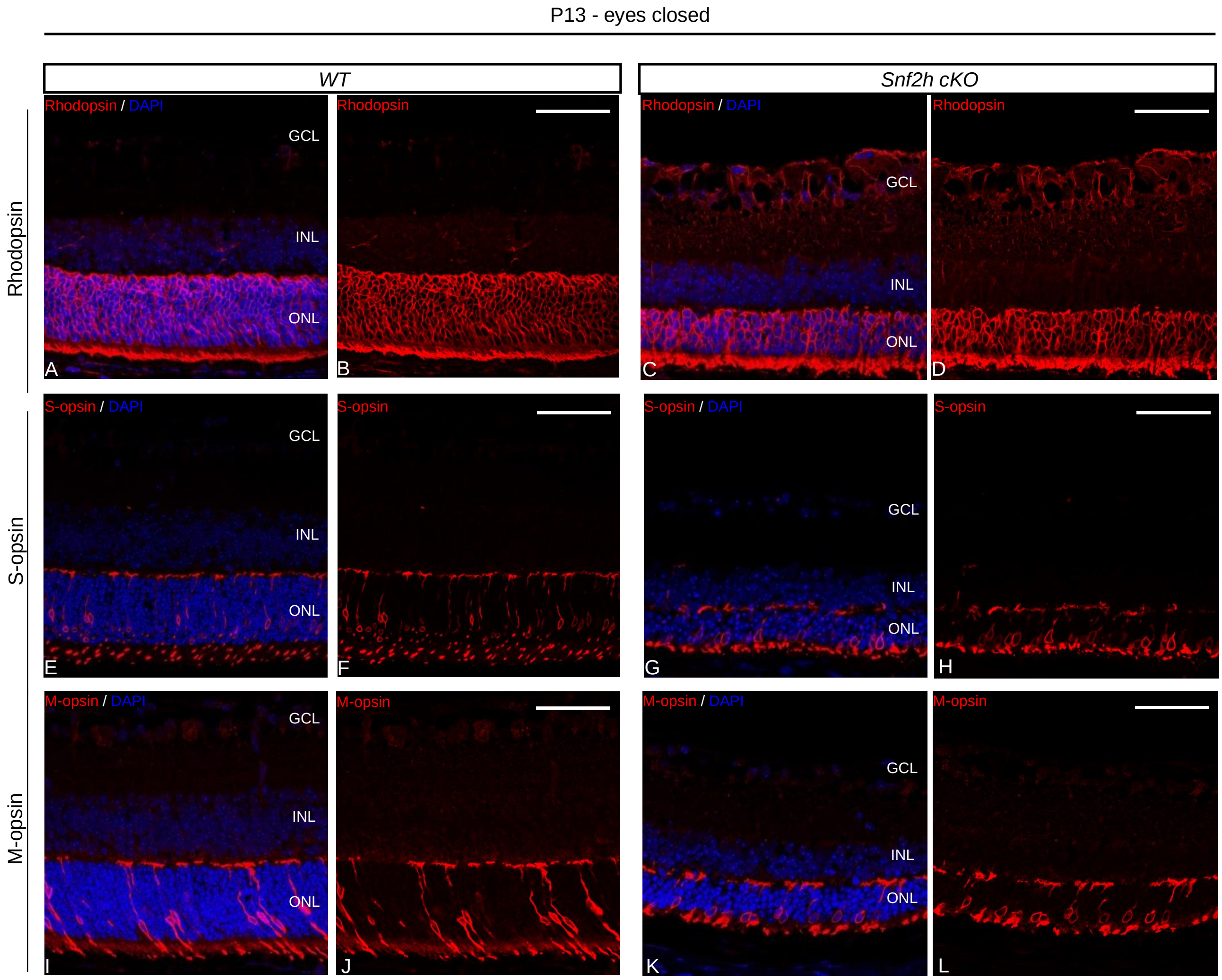

Supplementary Figure S6. Photoreceptor characterization at P13 (before eye opening).

Rod photoreceptors in the wild-type retina (A, B). The rod photoreceptors in Snf2h cKO were present (C, D). Although the number of rods was reduced, their cell shape and positioning was comparable with the wild-type controls. The same result was obtained with cone photoreceptor staining (E-H, I-L).

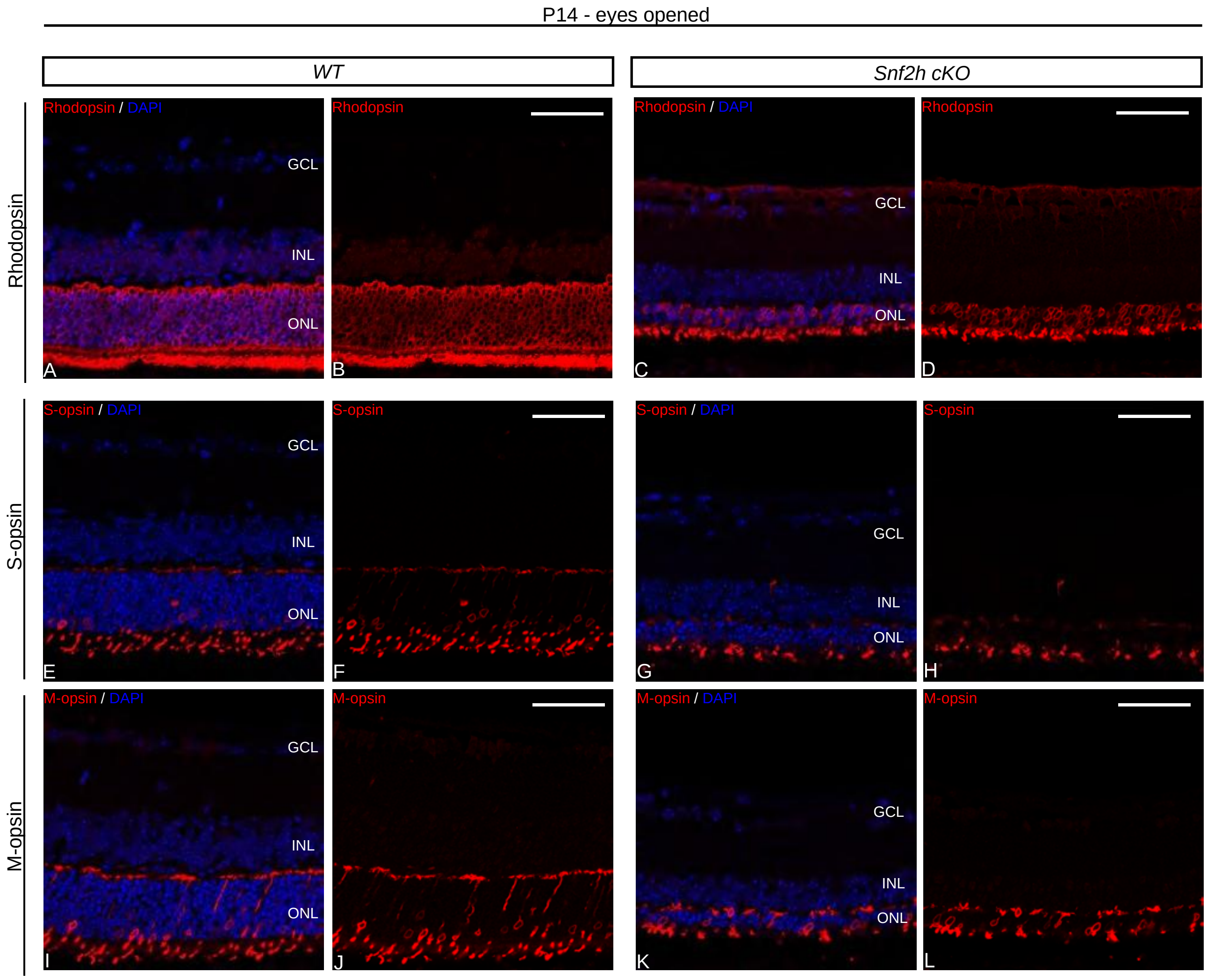

Supplementary Figure S7. Photoreceptor characterization at P14 (after eye opening).

The rod photoreceptors in Snf2h cKO were preserved, but their length was reduced by about a half compared with control, which was associated with reduced thickness of the ONL in Snf2h cKO (A-D). The cone photoreceptors exhibited an abnormal shape, the outer segments were reduced, and the photoreceptor protrusions detected in wild-type animals inside the OPL were missing in Snf2h cKO (E-H, I-L).

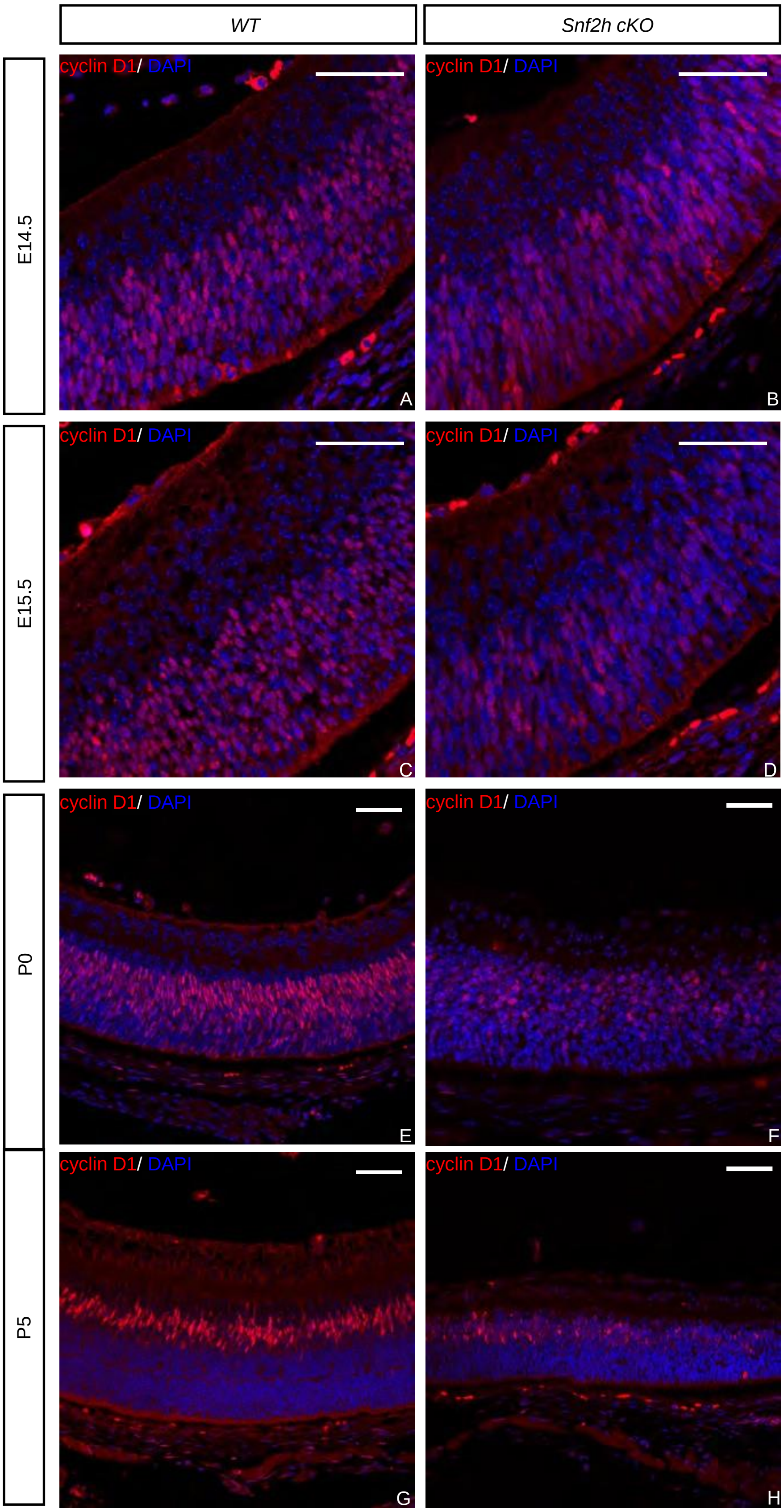

Supplementary Figre S8. Expression of cyclin D1 in Snf2h cKO during embryonic and postnatal stages.

Immunostaining for cyclin D1, required for G1/S transition during the cell cycle, in wild-type and Snf2h cKO. Differences in cyclin D1 immunoreactivity appeared already during embryonic development (A-D). A dramatic decrease in cyclin D1-positive cells and cyclin D1 expression level in Snf2h cKO compared with wild-type controls was observed at birth (E, F) and at P5 (G, H).

Supplementary Table S1. List of primary antibodies used for immunohistochemistry

Antigen	Host	Dilution	Retinal cell types	stage
Snf2h (Abcam)	Rabbit	1:500		Adult
Rhodopsin (Chemicon)	Mouse	1:200	Cone photoreceptor cells	Adult
S-opsin (Santa Cruz)	Goat	1:500	Cone photoreceptor cells	Adult
M-opsin (Santa Cruz)	Rabbit	1:500	Cone photoreceptor cells	Adult
Lhx2 (Santa Cruz)	Goat	1:1000	Müller glial cells	Adult
Chx10 (ThermoFisher Scientific)	Sheep	1:500	Bipolar cells	Adult
Oc2 (R&D Systems)	Sheep	1:500	Horizontal cells	Adult
Pax6 (Covance)	Rabbit	1:500	Amacrine cells	Adult
Brn3a (Dr. E. Turner)	Rabbit	1:4000	Retinal ganglion cells	Adult
Tbr2 (Abcam)	Rabbit	1:500	Retinal ganglion cells	Adult
Otx2 (Dr. Vaccarino)	Rabbit	1:500	Photoreceptor cells	Embryo
Crx (Santa Cruz)	Rabbit	1:100	Photoreceptor cells	Embryo
Blimp1 (Santa Cruz)	Rat	1:300	Photoreceptor cells	Embryo
Rxrγ (Santa Cruz)	Rabbit	1:3000	Photoreceptor cells	Embryo
cCas3 (Cell Signaling)	Rabbit	1:500		Embryo
Cyclin D1 (Santa Cruz)	Mouse	1:300		Embryo
pH3 (Santa Cruz)	Rabbit	1:800		Embryo
CENP-A (Cell Signaling)	Rabbit	1:500		Embryo

Supplementary Table S2. List of primers used for qRT-PCR

Name	Sequence (5'→3')
Atm-F	CCAGGGGAAGATGATGAAGA
Atm-R	TCGGCAGCTAAAGGACTCAT
Atr-F	GCTGTAGCGTCCTTTCGTTC
Atr-R	GGCTCATGCATAGCAGCATA
Casp3-F	GAGATGGCTTGCCAGAAGAT
Casp3-R	CCGTCCTTTGAATTTCTCCA
Casp9-F	GATGCTGTCCCCTATCAGGA
Casp9-R	CAGAATGCCATCCAAGGTCT
Cdkn2a-F	GCTCTGGCTTTCGTGAACAT
Cdkn2a-R	CGAATCTGCACCGTAGTTGA
CyclinA-F	CTGTCTCTTTACCCGGAGCA
CyclinA-R	AACGTTCACTGGCTTGTCTTC
CyclinB-F	GAGAAGGTGCCTGTGTGTGA
CyclinB-R	GGCTTGGAGAGGGATTATCA
CyclinE-F	CGTTACATGGCATCACAACA
CyclinE-R	GGTGCAACTTTGGAGGGTAG
CyclinG-F	GGCTTTGACACGGAGACATT
CyclinG-R	AGTCGCTTTCACAGCCAAAT
Mdm2-F	TGTGTGAGCTGAGGGAGATG
Mdm2-R	ATCCTGATCCAGGCAATCAC
p21-F	GTACTTCCTCTGCCCTGCTG
p21-R	TCTGCGCTTGGAGTGATAGA
p48-F	AACCAGGCCCAGAAGGTTAT
p48-R	CCTCTGGGGTCCACACTTTA
p53-F	CTAGCATTCAGGCCCTCATC
p53-R	ACTCCTCCATGGCAGTCATC
Gadd45b-F	CACCCTGATCCAGTCGTTCT
Gadd45b-R	TTGGCTTTTCCAGGAATCTG
Sfn-F	TGGCCCTGAACTTTTCAGTC
Sfn-R	GATGAGGGTGCTGTCCTTGT
UBB-F	ATGTGAAGGCCAAGATCCAG
UBB-R	TAATAGCCACCCCTCAGACG
Actb-F	GTCCACACCCGCCACCAGTTC
Actb-R	TTCTCCATGTCGTCCCAGTTG
Ubb-F	ATGTGAAGGCCAAGATCCAG
Ubb-R	TAATAGCCACCCCTCAGACG
Sdha-F	AAGGCAAATGCTGGAGAAGA
Sdha-R	TGGTTCTGCATCGACTTCTG
Gapdh-F	AACTTTGGCATTGTGGAAGG
Gapdh-R	ATCCACAGTCTTCTGGGTGG
